# Radiation increases COL1A1, COL3A1, and COL1A2 expression in breast cancer

**DOI:** 10.1515/med-2022-0436

**Published:** 2022-02-17

**Authors:** Guorong Yao, Kaiyue Zhao, Kaikai Bao, Jing Li

**Affiliations:** Department of Radiation Oncology, 1st Affiliated Hospital of Zhejiang University, 79# Qingchun Road, 310009 Hangzhou, China; Department of Radiology, The Affiliated Hospital of Hangzhou Normal University, 310015 Hangzhou, China; Department of Radiology, 1st People’s Hospital of Yuhang district, 310000 Hangzhou, China; Department of Nuclear Medicine, The Second Affiliated Hospital of Zhejiang University School of Medicine, 310009 Hangzhou, China

**Keywords:** radiotherapy, secondary cancer, breast cancer, Type I collagen, Type III collagen

## Abstract

**Background:**

Radiotherapy-associated secondary cancer is an important issue for the treatment of breast cancer (BCa). This study aimed to investigate the molecular mechanism and genetic risk factors for radiation-associated secondary diseases in BCa.

**Methods:**

The differentially expressed genes (DEGs) between preradiation and postradiation BCa samples in the GSE65505 dataset were obtained. The pathways related to the radiation-associated DEGs in the protein–protein interaction (PPI) network modules were identified. miRNAs targeted to the key genes in the PPI network were identified, and their association with BCa prognosis was analyzed.

**Results:**

A total of 136 radiation-associated DEGs preradiation and postradiation BCa samples were screened out. The PPI network consisted of a significant module that consisted of 21 upregulated DEGs that were associated with “hsa04512: ECM–receptor interaction,” “hsa04151: PI3K-Akt signaling pathway,” and “hsa04115: p53 signaling pathway.” Sixteen DEGs, including three collagen genes collagen type I alpha 1 chain (*COL1A1*), *COL3A1*, and *COL1A2*, were enriched in 17 radiation-associated pathways. The three genes were upregulated in BCa tissues compared with controls and were also elevated by radiation. They were targeted by *hsa*-*miR*-*29a*/*c*, and the expression levels of *hsa*-*miR*-*29a*/*c* were associated with a poor prognosis of BCa.

**Conclusions:**

The upregulation of *COL1A1*, *COL3A1*, and *COL1A2* might be genetic risk factors for radiation-associated secondary diseases in BCa.

## 1 Introduction

Breast cancer (BCa) is the most commonly diagnosed malignant cancer and the leading cause of cancer-related premature mortality in women worldwide [1]. About 2.1 million newly diagnosed cases of BCa were estimated in 2018 [1]. Epidemiologic studies have well established a series of risk factors for BCa, including family history, race, physical inactivity, obesity, genetic variants, and instability [2,3]. However, the treatment of BCa is still an important issue due to the high recurrence and mortality of this disease.

Adjuvant radiotherapy is the standard treatment for BCa patients that are undergoing breast-conservation surgery or mastectomy. Radiotherapy reduces BCa recurrence and death significantly [4,5,6]. Overall, radiotherapy reduces the 10 year risk of first recurrence from 35.0 to 19.3% and decreases the 15 year risk of death from 25.2 to 21.4% [4]. However, there are increasing evidence showing that radiotherapy causes secondary cancers and heart diseases decades later [7,8,9,10]. For instance, patients exposed to radiotherapy for prostate cancer and BCa had an over-time increased risk of bladder, colorectal, lung, and thyroid cancers compared with patients unexposed to radiotherapy [8,10]. In addition, the chest wall symptoms in BCa patients exposed to radiotherapy were worse than that of patients unexposed to radiotherapy [9]. Hodgkin lymphoma patients that were treated with chest radiotherapy have a high risk of BCa [11]. However, the molecular mechanisms of the pathogenesis of radiation-associated secondary cancers are not clear until now.

Research has pointed out that the smoking habit is a risk factor for radiation-associated secondary primary cancer [12]. In addition, there is evidence showing genetic susceptibility to radiation-associated secondary BCa [11]. Hodgkin lymphoma patients that had a high risk of radiation-associated secondary BCa had a higher polygenic risk score that was composed of the features of nine single-nucleotide polymorphisms [11]. For radiation-associated secondary cancers in patients with BCa, however, the molecular mechanism has not been reported until now.

For filling this gap, we performed this study to identify the radiation-associated genes in BCa. The identification of radiation-related genetic susceptibility and genetic risk factors would be of great value for better understanding the molecular mechanisms underlying the radiation-associated secondary cancers in patients with BCa.

## 2 Materials and methods

### 2.1 Microarray data

The mRNA expression dataset GSE65505 was downloaded from the National Center for Biotechnology Information (NCBI) Gene Expression Omnibus (GEO; http://www.ncbi.nlm.nih.gov/geo/). GSE65505 (GPL17586 platform, [HTA-2_0] Affymetrix Human Transcriptome Array 2.0 [transcript (gene) version]) consisted of 62 BCa tumor samples, including 29 paired preradiation and postradiation tumor samples: 6, 8, and 15 patients treated with 15 Gy, 18 Gy, and 21 Gy intensity-modulated radiotherapy. BCa samples were extracted from women (aged >55 years old) that had clinically node negative, estrogen receptors (ER)+ and/or PR+, Human epidermal growth factor receptor-2 (HER2−), T1 invasive carcinomas or low-intermediate grade *in situ* disease (≤2 cm).

### 2.2 Data processing and identification of radiation-associated genes

The series matrix files from paired preradiation and postradiation tumor samples (*n* = 58) were downloaded and preprocessed using the Limma package (version 3.34.0; https://bioconductor.org/packages/release/bioc/html/limma.html). Then the differentially expressed genes (DEGs) between paired preradiation and postradiation tumor samples were identified using the Limma package with the criteria of *p* < 0.05 and |log_2_(fold change, FC)| ≥ 0.585 (|FC| ≥ 1.5). The sample hierarchical clustering analysis was performed for DEGs using the pheatmap package (version 1.0.8; https://cran.r-project.org/package=pheatmap). The genes common to 15, 18, and 21 Gy radiotherapy-induced DEGs were identified using the Venn diagram analysis and were regarded as radiation-associated genes in BCa.

### 2.3 Enrichment analysis of radiation-associated genes

To illuminate the radiation-induced molecular functional changes in BCa, the Gene Ontology biological processes and Kyoto Encyclopedia of Genes and Genomes (KEGG) pathways that were associated with the radiation-associated genes DEGs were identified. Functional enrichment analysis was performed using the clusterprofiler package (https://github.com/GuangchuangYu/clusterProfiler). The threshold for selecting the significant biological processes and pathways were set at adjusted *p* value <0.05 and gene count ≥2.

### 2.4 Construction of protein–protein interaction network (PPI) of radiation-associated DEGs

The interactions between the products of radiation-associated DEGs in the GSE65505 dataset were predicted in the STRING database (Version: 10.0, http://www.string-db.org/) with the default parameter (score >0.4). Then the PPI network of radiation-associated genes was constructed using the Cytoscape software (version 3.4.0, http://chianti.ucsd.edu/cytoscape-3.4.0/). The important module in the PPI network (score >5) was identified using the MCODE plugin in Cytoscape. Radiation-associated genes that were included in the important modules were subjected to the functional enrichment analysis using the clusterprofiler package. The significant pathways that were associated with the genes in important module were selected using the same criteria as mentioned above.

### 2.5 Identification of BCa-associated pathways

To select the key pathways related to the radiation-associated DEGs in the important module, the BCa-associated pathways in Comparative Toxicogenomics Database (CTD, http://ctdbase.org/) were identified using the searching term of “breast neoplasm.” The items overlapped between the CTD database and the KEGG pathways were regarded as the key pathways in BCa, and the enriched genes were regarded as the key radiation-associated DEGs in BCa.

### 2.6 Construction of miRNA–target network

The miRNA–target pairs of the aforementioned key radiation-associated DEGs in BCa were predicted using the Webgestal online tool [13] using the overrepresentation enrichment analysis methods. Then the miRNA–target pairs with the *p* value of less than 0.05 were selected and used for the construction of miRNA–target network. The network was visualized using the Cytoscape software.

### 2.7 Identification of prognosis-associated genes and miRNAs in BCa

To investigate the association of genes and miRNAs with the prognosis of BCa, the correlation of aforementioned radiation-associated genes and miRNAs in the miRNA–target network with the overall survival (OS) of patients with breast invasive carcinoma (BRCA) in the Cancer Genome Atlas (TCGA) were analyzed using the user-friendly and interactive web resource of UALCAN (http://ualcan.path.uab.edu/index.html). Significantly difference was identified using the criterion of logRank *p* < 0.05.

## 3 Results

### 3.1 Identification of radiation-associated genes in BCa in GSE65505

**Figure 1:**
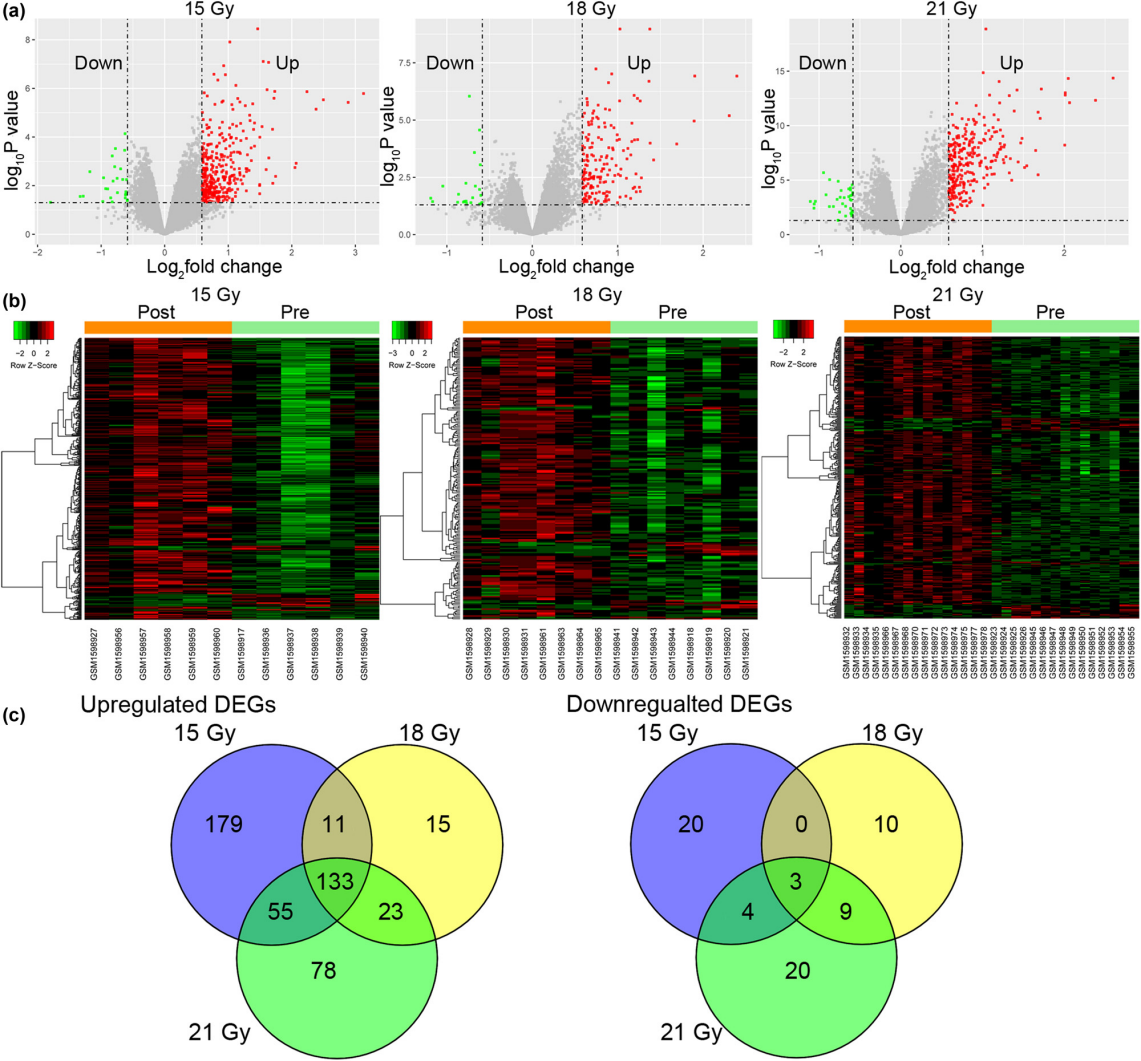
The statistics and clustering of the DEGs in BCa preradiation and postradiation: (a) the volcano plot of the DEGs in BCa samples between preradiotherapy and postradiotherapy (15 Gy, 18 Gy, and 21 Gy), (b) the hierarchical clustering analysis of DEGs, and (c) the Venn diagram of the upregulated and downregulated radiation-associated genes in BCa in the GSE65505 dataset.

A total of 405, 204, and 325 DEGs were identified in BCa tumor samples receiving 15 Gy, 18 Gy, and 21 Gy intensity-modulated radiotherapy in the GSE65505 dataset, respectively (Figure 1a). Hierarchical clustering analysis demonstrated the distinct expression profiles of these DEGs in samples preradiation and postradiation (Figure 1b). Venn diagram analysis showed that common 133 DEGs were upregulated by the three radiotherapies, and only three DEGs were commonly downregulated (Figure 1c). Then, 136 radiation-associated DEGs in BCa were identified in this study (Table A1).

### 3.2 Enrichment analysis of upregulated radiation-associated DEGs in BCa

The functional enrichment analysis of the 136 radiation-associated DEGs indicated that these genes were enriched into 563 biological processes that were associated with extracellular matrix (ECM) organization, inflammatory response, response to metal ion, neutrophil-mediated immunity, and cell chemotaxis, and 38 KEGG pathways including advanced glycation end-receptor for dvanced glycation end products (AGE-RAGE) signaling pathway in diabetic complication, p53 signaling pathway, PI3K-Akt signaling pathway, and ECM–receptor interaction. The top ten biological processes and KEGG pathways are shown in Table A2.

### 3.3 PPI network of radiation-associated DEGs in BCa

**Figure 2:**
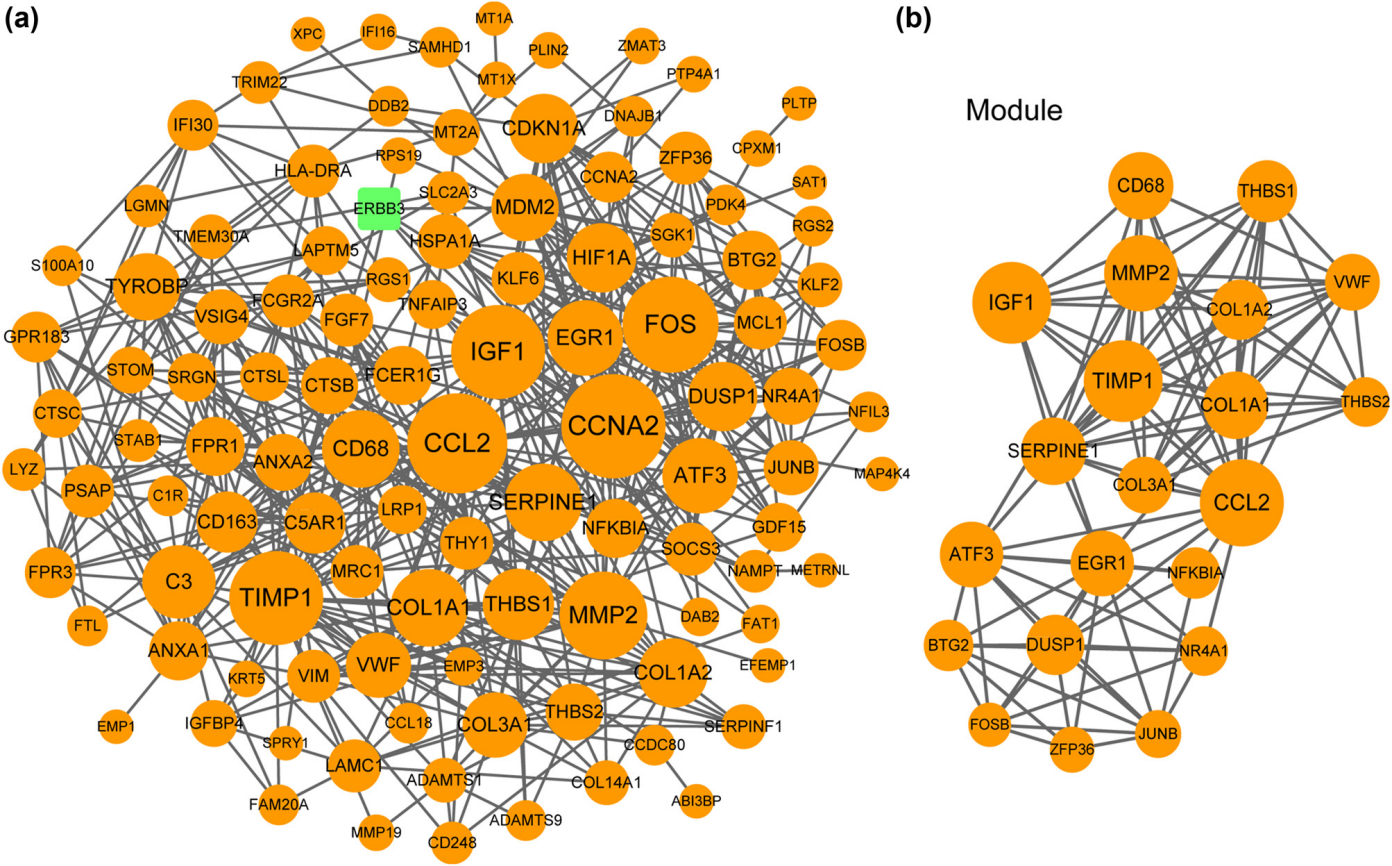
The PPI network and the important module of the radiation-associated genes in BCa: (a) the PPI network consisted of 111 upregulated and 1 downregulated radiation-associated genes in BCa and (b) the important module (score = 9.9, 21 nodes) identified using MCODE. Node size indicates the interaction degree. The higher the degree, the larger the node size is. The one downregulated radiation-associated gene is presented by blue square.

The PPI network that derived from the 136 radiation-associated genes was composed of 112 nodes (products of genes) and 529 lines (interaction pairs; Figure 2a). One module of a score of 9.9 and 21 upregulated radiation-associated DEGs was identified from the PPI network (Figure 2b). The interaction degree of these nodes is shown in Table 1. The insulin like growth factor 1 (*IGF1*), matrix metallopeptidase 2 (*MMP2*), collagen type I alpha 1 chain (*COL1A1*), *COL1A2*, and *COL3A1* gene had the interaction degrees of 30, 27, 22, 18, and 16, respectively.

**Table 1:** The list and interaction degree of the 21 upregulated radiation-associated genes in the module of the PPI network of breast cancer

Nodes	Regulation	Degree	Nodes	Regulation	Degree
*CCL2*	UP	33	*DUSP1*	UP	18
*TIMP1*	UP	30	*COL3A1*	UP	16
*IGF1*	UP	30	*VWF*	UP	16
*MMP2*	UP	27	*BTG2*	UP	13
*COL1A1*	UP	22	*NFKBIA*	UP	13
*CD68*	UP	22	*THBS2*	UP	12
*SERPINE1*	UP	22	*NR4A1*	UP	12
*EGR1*	UP	21	*JUNB*	UP	11
*ATF3*	UP	21	*ZFP36*	UP	10
*THBS1*	UP	19	*FOSB*	UP	9
*COL1A2*	UP	18			

CCL2: C-C motif chemokine ligand 2; MMP2: matrix metallopeptidase 2; COL1A1: collagen type I alpha 1 Chain; SERPINE1: serpin family E member 1; EGR1: early growth response 1; COL3A1: collagen type III alpha 1 chain; THBS1: thrombospondin 1; VWF: von Willebrand factor; THBS2: thrombospondin 2; NFKBIA: nuclear factor kappa B inhibitor alpha; FOSB: FosB proto-oncogene, AP-1 transcription factor subunit; DUSP1: dual specificity phosphatase 1.

### 3.4 Pathways that related to radiation-associated genes

The 21 upregulated radiation-associated DEGs in the module were associated with 19 KEGG pathways such as “hsa04933: AGE-RAGE signaling pathway in diabetic complications,” “hsa04512: ECM–receptor interaction,” “hsa04151: PI3K-Akt signaling pathway,” and “hsa04115: p53 signaling pathway” (Table 2). The function properties of the 21 radiation-associated genes were basically in line with the 136 radiation-associated DEGs in BCa. Among the 19 pathways, 17 pathways that were common to BCa-related pathways in the CTD database were regarded as the key radiation-associated pathways in BCa. Moreover, 16 upregulated radiation-associated DEGs (including *IGF1*, *MMP2*, *COL1A1*, *COL3A1*, and *ZFP36*) were involved in the 17 key radiation-associated pathways (Table 2).

**Table 2:** The list of top 10 biological processes and KEGG pathways related to the radiation-associated genes in the module of the PPI network of breast cancer

ID	Description	Count	*p* value	*p*. adjust	Gene symbol
*hsa04933	AGE-RAGE signaling pathway in diabetic complications	7	2.53 × 10^−9^	2.71 × 10^−7^	*CCL2*, *MMP2*, *COL1A1*, *SERPINE1*, *EGR1*, *COL1A2*, and *COL3A1*
*hsa04512	ECM–receptor interaction	5	1.91 × 10^−6^	1.02 × 10^−4^	*COL1A1*, *THBS1*, *COL1A2*, *VWF*, and *THBS2*
*hsa04510	Focal adhesion	6	6.19 × 10^−6^	2.21 × 10^−4^	*IGF1*, *COL1A1*, *THBS1*, *COL1A2*, *VWF*, and *THBS2*
hsa04926	Relaxin signaling pathway	5	1.26 × 10^−5^	3.05 × 10^−4^	*MMP2*, *COL1A1*, *COL1A2*, *COL3A1*, and *NFKBIA*
*hsa04151	PI3K-Akt signaling pathway	7	1.43 × 10^−5^	3.05 × 10^−4^	*IGF1*, *COL1A1*, *THBS1*, *COL1A2*, *VWF*, *THBS2*, and *NR4A1*
*hsa05205	Proteoglycans in cancer	5	1.17 × 10^−4^	2.08 × 10^−3^	*IGF1*, *MMP2*, *COL1A1*, *THBS1*, and *COL1A2*
*hsa04611	Platelet activation	4	2.16 × 10^−4^	3.20 × 10^−3^	*COL1A1*, *COL1A2*, *COL3A1*, and *VWF*
*hsa05144	Malaria	3	2.40 × 10^−4^	3.20 × 10^−3^	*CCL2*, *THBS1*, and *THBS2*
*hsa04115	p53 signaling pathway	3	7.04 × 10^−4^	8.37 × 10^−3^	*IGF1*, *SERPINE1*, and *THBS1*
hsa05165	Human papillomavirus infection	5	1.05 × 10-3	1.13 × 10-2	*COL1A1*, *THBS1*, *COL1A2*, *VWF*, and *THBS2*
*hsa04657	IL-17 signaling pathway	3	1.53 × 10^−3^	1.40 × 10^−2^	*CCL2*, *NFKBIA*, and *FOSB*
*hsa04974	Protein digestion and absorption	3	1.58 × 10^−3^	1.40 × 10^−2^	*COL1A1*, *COL1A2*, and *COL3A1*
*hsa05142	Chagas disease (American trypanosomiasis)	3	1.93 × 10^−3^	1.48 × 10^−2^	*CCL2*, *SERPINE1*, and *NFKBIA*
*hsa05146	Amoebiasis	3	1.93 × 10^−3^	1.48 × 10^−2^	*COL1A1*, *COL1A2*, and *COL3A1*
*hsa04066	HIF-1 signaling pathway	3	2.34 × 10^−3^	1.67 × 10^−2^	*TIMP1*, *IGF1*, and *SERPINE1*
*hsa04668	TNF signaling pathway	3	2.53 × 10^−3^	1.69 × 10^−2^	*CCL2*, *NFKBIA*, and *JUNB*
*hsa04380	Osteoclast differentiation	3	3.69 × 10^−3^	2.32 × 10^−2^	*NFKBIA*, *JUNB*, and *FOSB*
*hsa05219	Bladder cancer	2	4.55 × 10^−3^	2.62 × 10^−2^	*MMP2* and *THBS1*
*hsa05418	Fluid shear stress and atherosclerosis	3	4.65 × 10^−3^	2.62 × 10^−2^	*CCL2*, *MMP2*, and *DUSP1*

*Notes the overlapping KEGG pathways between the comparative toxicogenomics database and that related to the 21 radiation-sensitive genes.

### 3.5 Construction of the radiation-associated miRNA–target network in BCa

**Figure 3:**
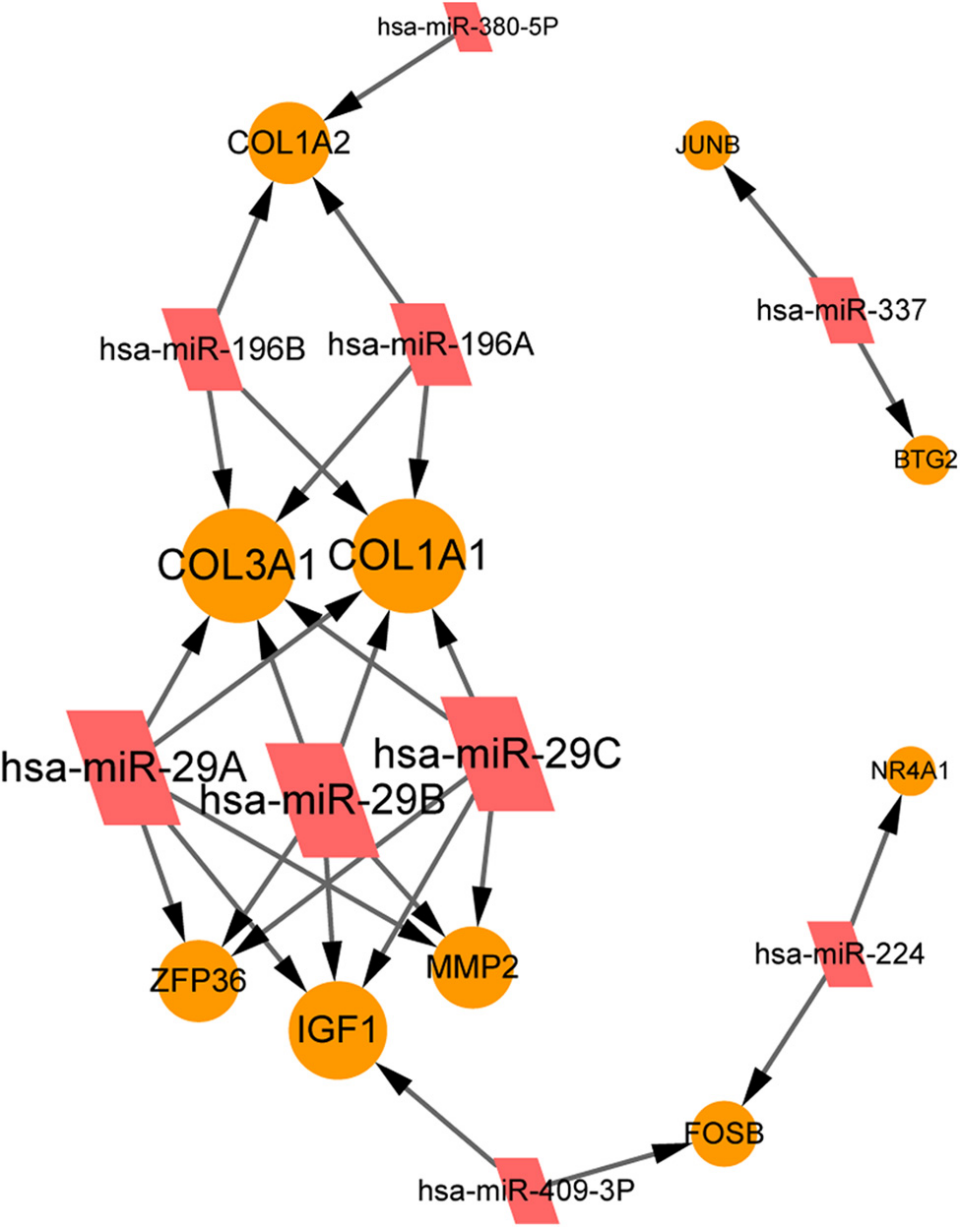
The miRNA–target regulatory network involving the radiation-associated genes in the key pathways related to BCa. Node size indicates the interaction degree. The higher the degree, the larger the node size is. The miRNA–target interaction pairs were identified using the Webgestal online tool. The miRNA–target pairs with a *p* < 0.05 are used for the construction of the miRNA–target network.

The miRNA–target pairs of the aforementioned 16 radiation-associated DEGs in BCa were predicted, and the corresponding radiation-associated miRNA–target network was constructed (Figure 3). This miRNA–target network consisted of ten radiation-associated DEGs, including *IGF1*, *MMP2*, *COL1A1*, *COL3A1*, and *ZFP36*, and nine miRNAs, including *hsa*-*miR*-*2*9a, *hsa*-*miR*-*29b*, *hsa*-*miR*-*29c*, *hsa*-*miR*-*196a*, and *hsa*-*miR*-*196b*. In this network, five radiation-associated DEGs, including *IGF1*, *MMP2*, *COL1A1*, *COL3A1*, and *ZFP36*, were simultaneously regulated by *hsa*-*miR*-*29a*, *hsa*-*miR*-*29b*, and *hsa*-*miR*-*29c*. *hsa*-*miR*-*196a* and *hsa*-*miR*-*196b* both targeted to three collagen genes, including *COL1A1*, *COL3A1*, and *COL1A2*. The other four radiation-associated DEGs, including JunB proto-oncogene (*JUNB*), BTG antiproliferation factor 2 (*BTG2*), FosB proto-oncogene, AP-1 transcription factor subunit (*FOSB*), and nuclear receptor subfamily 4 group A member 1 (*NR4A1*), were regulated by one or two miRNAs, respectively (Figure 3).

### 3.6 The association of radiation-associated genes with the prognosis of BCa

**Figure 4:**
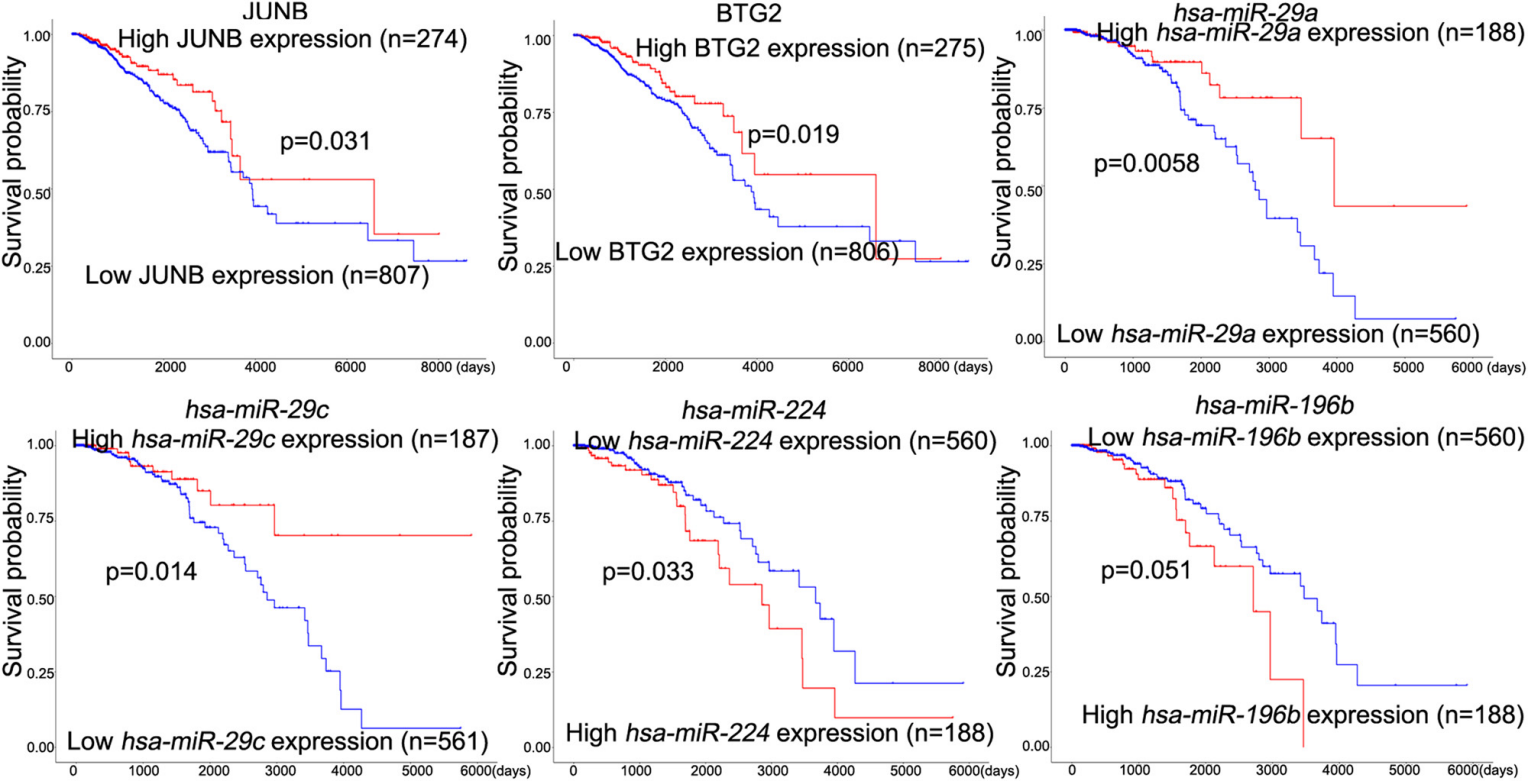
The survival analysis of genes and miRNAs in the miRNA–target regulatory network. Survival analysis was performed using the UALCAN online tool and based on the TCGA cohort (breast invasive carcinoma).

Based on the analysis in the UALCAN database, we found that two genes, BTG2 and JUNB, and four miRNAs, including *hsa*-*miR*-*29a*, *hsa*-*miR*-*29c*, *hsa*-*miR*-*224*, and *hsa*-*miR*-*196b*, were significantly associated with the prognosis in BCa (Figure 4). The high expression levels of *JUNB* (*p* = 0.031), *BTG2* (*p* = 0.019), *hsa*-*miR*-*29a* (*p* = 0.0058), and *hsa*-*miR*-*29c* (*p* = 0.014) as well as the low expression levels of *hsa*-*miR*-*224* (*p* = 0.033) and *hsa*-*miR*-*196b* (*p* = 0.051) were associated with a low survival probability in the TCGA BRCA patients (Figure 4).

**Figure 5:**
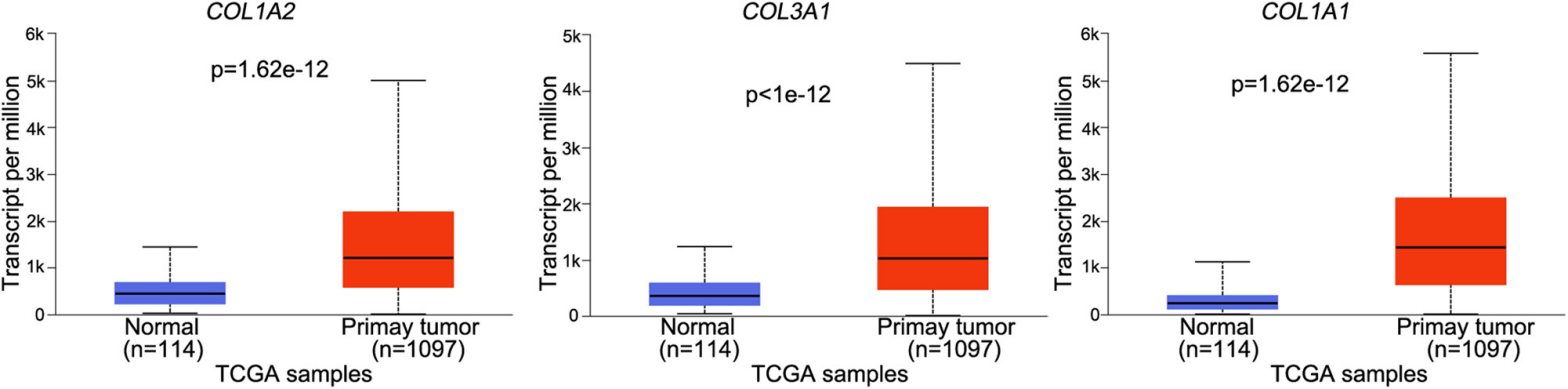
The expression of the *COL1A1*, *COL3A1*, and *COL1A2* genes in the TCGA breast cancer tumor and normal control samples. The statistical differences were analyzed using the UALCAN online tool. Data was expressed as median and range (minimum to maximum).

Besides, we found three genes, including *COL1A1*, *COL3A1*, and *COL1A2*, were significantly upregulated in TCGA BRCA tumor samples compared with normal controls (Figure 5), whereas the other seven genes were not significantly changed in BCa tumor samples (data now shown).

## 4 Discussion

The increasing incidence of radiation-associated secondary cancers is challenging the treatment strategies for human solid cancers. Based on the tumorigenicity [11], we speculated that the mechanisms responsible for radiation-associated secondary cancers involve the dysregulation of a cluster of genetic factors. As expected, we identified that the upregulation of three collagen genes (*COL1A1*, *COL3A1*, and *COL1A2*), *BTG2*, and *JUNB* might be responsible for the radiation-associated secondary cancers in patients with BCa.

COL1A1 and COL3A1, respectively, encodes the α1 chain of Type I and III collagen, both of which are the fibril-forming collagens [14,15]. Type I and Type III procollagens are crucial components of ECM, which is important for tumor microenvironment [14]. They act important roles in the proliferation and/or metastasis of cancer cells. Previous study showed that the Type I and Type III procollagens were elevated in human scirrhous carcinoma of breast with a spatial specificity [16]. *In situ* hybridization showed that the expression levels of the Type I and Type III procollagens were decreased with the increased distance between the fibroblasts and tumor cells [16]. The upregulation and association of *COL1A1*, *COL3A1*, and *COL1A2* with collagen prolyl-4-hydroxylase α subunit 2 (*P4HA2*) had been reported in BCa [17,18,19]. In addition, the other factors that related to ECM or collagens, including *MMP2*, *CCL2*, and tissue inhibitor of metalloproteinase-1 (*TIMP1*), were also identified to be upregulated in BCa tissues compared with control [18]. Here, in our present study, we identified that the exposure to radiotherapy elevated the expression of *COL1A1*, *COL3A1*, and *COL1A2* in BCa tissues rather than decreased them. According to the above studies, we assumed that the consistent upregulation of the three genes might of great value for exploring the development of radiation-associated secondary nonbreast diseases in BCa.

There are tremendous evidence showing that *COL1A1*, *COL3A1*, *COL1A2*, or *P4HA2* promotes the metastasis of BCa and contributes to a poor survival in patients with ER+ BCa [14,18]. By contrast, the inhibition of *COL1A1* or *P4HA2* counteracted the metastasis and suppressed the proliferation of the BCa cells [14,18]. The study by Srour et al. [20] showed that *MMP2*, *COL1A1*, *COL3A1*, and *COL1A2* had lower expression levels in lymph node metastasis compared with triple-negative BCa (TNBC). This finding was in line with the fact that from Gan. [16] who showed that the expression of the Type I and Type III procollagens were decreased with the increased distance between fibroblasts and tumor cells. The interesting evidence was that the elevated *COL1A1* expression in BCa had been reported to be correlated with a better response to cisplatin-based chemotherapy [14]. A recent study by Liu et al. [21] showed that there was an opposite conclusion that *COL1A1* expression was negatively correlated with radiosensitivity in cervical cancer. Liu et al. [21] reported that the *COL1A1* activation could inhibit X-ray radiation-induced apoptosis in cervical cancer. Our present study showed that *COL1A1* and *COL3A1* were targeted by *hsa-miR-29a/b/c* that were decreased in radio-resistant nasopharyngeal carcinoma cells [22]. The expression of *COL1A1* exerted a radio-resistance effect in nasopharyngeal carcinoma cells [22]. Accordingly, we suspected that the consistent radiation-associated upregulation of the *COL1A1*, *COL3A1*, and *COL1A2* genes in BCa tissues might be used as genetic susceptibility and risk factors for radio-resistance. This might be related to the radiation-associated secondary nonbreast diseases in patients with BCa.

The association of *hsa-miR-29* family members with tumorigenesis and drug-resistance in BCa cells had been proven previously [23,24,25]. *hsa-miR-29a* acts an oncogenic role [23,24,26], whereas the *hsa-miR-29c* exhibits a tumor suppressor role in BCa [25,26]. However, we found that the low expression levels of *hsa-miR-29a/c* were associated with a good survival outcome of BCa. Also, both of them targeted to the upregulated *COL1A1* and *COL3A1* genes. These results suggested that the predicted miRNA–mRNA regulatory networks, including *hsa-miR-29a/c*–*COL1A1*/*COL3A1*, might have crucial roles in the progression of BCa and the development of radiation-associated secondary diseases.

## 5 Conclusion

In summary, this present study showed that radiotherapy significantly upregulated the expression of *COL1A1*, *COL3A1*, and *COL1A2* genes in BCa tumor tissues. The upregulation of them might be risk factors for radiation-associated secondary nonbreast diseases in patients with BCa. However, the association of *COL1A1*, *COL3A1*, and *COL1A2* upregulation with radiation-associated secondary diseases should be validated using large cohort trials.

## Abbreviations

BCa: breast cancer
BRCA: breast invasive carcinoma
CTD: comparative Toxicogenomics Database
DEGs: differentially expressed genes
ECM: extracellular matrix
GEO: gene expression omnibus
IGF1: insulin like growth factor 1
KEGG: Kyoto Encyclopedia of Genes and Genomes
MMP2 : matrix metallopeptidase 2
OS: overall survival
PPI: protein–protein interaction
*TIMP1*
: tissue inhibitor of metalloproteinase-1
TNBC: triple-negative breast cancer

## Appendix

**Table A1:** The list of the radiation-associated genes in breast cancer (BCa) in the GSE65505 dataset

Gene symbol			
*EGR1*	*HSPA1A*	*COL1A2*	*FCER1G*
*ZFP36*	*S100A10*	*SNORD99*	*C1R*
*FOS*	*HIF1A*	*SNORD114-3*	*COL1A1*
*MDM2*	*PTP4A1*	*CD68*	*SGK1*
*FOSB*	*MMP2*	*COL14A1*	*CTSC*
*CCL2*	*SOCS3*	*LGMN*	*PSAP*
*MT1A*	*PDK4*	*JUN*	*VWF*
*LYZ*	*RGS2*	*COL3A1*	*XPC*
*ANXA1*	*MT1M*	*ABI3BP*	*IGF1*
*RASD1*	*NAMPT*	*THBS2*	*FCGR2C*
*TNFAIP3*	*ADAMTS9*	*MAP4K4*	*TMEM30A*
*MT2A*	*GEM*	*NR4A1*	*ATF3*
*CCN1*	*HLA-DRA*	*LRP1*	*SAMHD1*
*SERPINE1*	*SNORD92*	*EMP3*	*FPR1*
*MT1X*	*MT1JP*	*IL6-AS1*	*TYROBP*
*KLF6*	*LAPTM5*	*MMP19*	*C5AR1*
*SLC2A3*	*MCL1*	*SERPINF1*	*CPXM1*
*SRGN*	*PLTP*	*CTSL*	*FGF7*
*KRT5*	*ANXA2*	*ZMAT3*	*H19*
*DUSP1*	*FAT1*	*DAB2*	*SLIT3*
*NFKBIA*	*NFIL3*	*IFI30*	*VGLL3*
*GPR183*	*CD163*	*XGY2*	*SNORD114-21*
*EMP1*	*VIM*	*KLF2*	*THY1*
*THBS1*	*TRIM22*	*PTCSC1*	*EDA2R*
*CCDC80*	*EFEMP1*	*GDF15*	*PLIN2*
*YBX3*	*CD248*	*SPRY1*	*METRNL*
*STOM*	*FTL*	*FAM20A*	*VSIG4*
*SNORD114-14*	*CDKN1A*	*ADAMTS1*	*DRAM1*
*IFI16*	*SNORD114-12*	*ECSCR*	*CTSB*
*RPS19*	*DNAJB1*	*C3*	*STAB1*
*SAT1*	*RGS1*	*IGFBP4*	*CCL18*
*BTG2*	*SNORD114-1*	*FPR3*	**POTEKP*
*NEAT1*	*JUNB*	*LAMC1*	**ERBB3*
*DDB2*	*MRC1*	*TIMP1*	**CYP4X1*

*The three downregulated radiation-sensitive genes.

**Table A2:** The top 15 gene ontology biological processes and the KEGG pathways related to the upregulated radiation-associated genes in breast cancer (BCa)

ID	Description	Count	*P* value	*P* adjusted	Gene symbol
GO:0030198	Extracellular matrix organization	18	1.18 × 10^−10^	2.15 × 10^−7^	*SERPINE1*, *THBS1*, *MMP2*, *COL1A2*, *TIMP1*, *COL1A1*, et al.
GO:0043062	Extracellular structure organization	19	1.42 × 10^−10^	2.15 × 10^−7^	*SERPINE1*, *THBS1*, *MMP2*, *COL1A2*, *TIMP1*, *COL1A1*, et al.
GO:0071674	Mononuclear cell migration	9	1.46 × 10^−8^	1.48 × 10^−5^	*SERPINE1*, *DUSP1*, *THBS1*, *RPS19*, *C5AR1*, *CCL18*, et al.
GO:0050727	Regulation of inflammatory response	17	5.81 × 10^−8^	4.41 × 10^−5^	*SERPINE1*, *NEAT1*, *SOCS3*, *SERPINF1*, *IGF1*, *CCL18*, et al.
GO:0070997	Neuron death	14	1.77 × 10^−7^	7.54 × 10^−5^	*EGR1*, *FOS*, *CCL2*, *BTG2*, *LRP1*, *SERPINF1*, *ERBB3*, et al.
GO:0002683	Negative regulation of immune system process	16	1.78 × 10^−7^	7.54 × 10^−5^	*ZFP36*, *THBS1*, *COL3A1*, *FCER1G*, *THY1*, *VSIG4*, et al.
GO:0071276	Cellular response to cadmium ion	6	2.16 × 10^−7^	7.54 × 10^−5^	*FOS*, *MT1A*, *MT2A*, *MT1X*, *MT1M*, *JUN*
GO:0031589	Cell-substrate adhesion	14	2.18 × 10^−7^	7.54 × 10^−5^	*SERPINE1*, *THBS1*, *COL3A1*, *MAP4K4*, *COL1A1*, et al.
GO:0030099	Myeloid cell differentiation	15	2.58 × 10^−7^	7.54 × 10^−5^	*ZFP36*, *THBS1*, *HIF1A*, *JUNB*, *KLF2*, *FCER1G*, et al.
GO:0010038	Response to metal ion	14	3.06 × 10^−7^	7.54 × 10^−5^	*FOSB*, *THBS1*, *HIF1A*, *JUNB*, *JUN*, *SERPINF1*, et al.
hsa04933	AGE-RAGE signaling pathway in diabetic complications	8	1.03 × 10^−5^	9.25 × 10^−4^	*EGR1*, *CCL2*, *SERPINE1*, *MMP2*, *COL1A2*, *JUN*, et al.
hsa04115	p53 signaling pathway	7	1.05 × 10^−5^	9.25 × 10^−4^	*MDM2*, *SERPINE1*, *THBS1*, *DDB2*, *IGF1*, et al.
hsa04151	PI3K-Akt signaling pathway	14	2.02 × 10^−5^	1.18 × 10^−3^	*THBS1*, *COL1A2*, *THBS2*, *COL1A1*, *IGF1*, *ERBB3*, et al.
hsa05205	Proteoglycans in cancer	10	6.11 × 10^−5^	2.03 × 10^−3^	*THBS1*, *MMP2*, *COL1A1*, *IGF1*, *ERBB3*, et al.
hsa04380	Osteoclast differentiation	8	6.22 × 10^−5^	2.03 × 10^−3^	*FOS*, *FOSB*, *JUNB*, *JUN*, *FCGR2C*, *TYROBP*, et al.
hsa05150	Staphylococcus aureus infection	7	6.92 × 10^−5^	2.03 × 10^−3^	*FPR3*, *C1R*, *FCGR2C*, *FPR1*, *C5AR1*, et al.
hsa05140	Leishmaniasis	6	1.62 × 10^−4^	3.58 × 10^−3^	*FOS*, *NFKBIA*, *HLA*-*DRA*, *JUN*, *C3*, *FCGR2C*
hsa04612	Antigen processing and presentation	6	1.74 × 10^−4^	3.58 × 10^−3^	*HSPA1A*, *HLA*-*DRA*, *LGMN*, *CTSL*, *IFI30*, *CTSB*
hsa04668	TNF signaling pathway	7	1.83 × 10^−4^	3.58 × 10^−3^	*FOS*, *CCL2*, *SOCS3*, *JUNB*, *JUN*, et al.
hsa04145	Phagosome	8	2.07 × 10^−4^	3.64 × 10^−3^	*THBS1*, *THBS2*, *CTSL*, *C3*, *C1R*, *FCGR2C*, et al.

## References

[1] Bray F, Ferlay J, Soerjomataram I, Siegel RL, Torre LA, Jemal A. Global cancer statistics 2018: GLOBOCAN estimates of incidence and mortality worldwide for 36 cancers in 185 countries. CA Cancer J Clin. 2018;68(6):394–424. 10.3322/caac.21492 30207593

[2] Paz M, de Alencar M, Gomes JA, Da CMK, Islam MT, Ali ES, et al. Correlations between risk factors for breast cancer and genetic instability in cancer patients-a clinical perspective study. Front Genet. 2017;8:236. 10.3389/fgene.2017.00236 PMC5821102 29503660

[3] Coughlin SS. Epidemiology of breast cancer in women. Adv Exp Med Biol. 2019;1152:9–29. 10.1007/978-3-030-20301-6_2 31456177

[4] Darby S, McGale P, Correa C, Taylor C, Arriagada R, Clarke M, et al. Effect of radiotherapy after breast-conserving surgery on 10 year recurrence and 15 year breast cancer death: meta-analysis of individual patient data for 10,801 women in 17 randomised trials. Lancet. 2011;378(9804):1707–16. 10.1016/S0140-6736(11)61629-2 PMC3254252 22019144

[5] Wang SL, Fang H, Song YW, Wang WH, Hu C, Liu YP, et al. Hypofractionated versus conventional fractionated postmastectomy radiotherapy for patients with high-risk breast cancer: a randomised, non-inferiority, open-label, phase 3 trial. Lancet Oncol. 2019;20(3):352–60. 10.1016/S1470-2045(18)30813-1 30711522

[6] Klein J, Tran W, Watkins E, Vesprini D, Wright FC, Look HN, et al. Locally advanced breast cancer treated with neoadjuvant chemotherapy and adjuvant radiotherapy: a retrospective cohort analysis. BMC Cancer. 2019;19(1):306. 10.1186/s12885-019-5499-2 PMC6448234 30943923

[7] Taylor C, Correa C, Duane FK, Aznar MC, Anderson SJ, Bergh J, et al. Estimating the risks of breast cancer radiotherapy: evidence from modern radiation doses to the lungs and heart and from previous randomized trials. J Clin Oncol. 2017;35(15):1641–9. 10.1200/JCO.2016.72.0722 PMC5548226 28319436

[8] Wallis CJ, Mahar AL, Choo R, Herschorn S, Kodama RT, Shah PS, et al. Second malignancies after radiotherapy for prostate cancer: systematic review and meta-analysis. BMJ. 2016;352:i851. 10.1136/bmj.i851 PMC4775870 26936410

[9] Velikova G, Williams LJ, Willis S, Dixon JM, Loncaster J, Hatton M, et al. Quality of life after postmastectomy radiotherapy in patients with intermediate-risk breast cancer (SUPREMO): 2 year follow-up results of a randomised controlled trial. Lancet Oncol. 2018;19(11):1516–29. 10.1016/S1470-2045(18)30515-1 30337220

[10] Grantzau T, Overgaard J. Risk of second non-breast cancer among patients treated with and without postoperative radiotherapy for primary breast cancer: a systematic review and meta-analysis of population-based studies including 522,739 patients. Radiother Oncol. 2016;121(3):402–13. 10.1016/j.radonc.2016.08.017 27639892

[11] Opstal-van WA, de Haan HG, Hauptmann M, Schmidt MK, Broeks A, Russell NS, et al. Genetic susceptibility to radiation-induced breast cancer after Hodgkin lymphoma. Blood. 2019;133(10):1130–9. 10.1182/blood-2018-07-862607 PMC6405334 30573632

[12] DiMarzio P, Peila R, Dowling O, Timony DM, Balgobind A, Lee LN, et al. Smoking and alcohol drinking effect on radiotherapy associated risk of second primary cancer and mortality among breast cancer patients. Cancer Epidemiol. 2018;57:97–103. 10.1016/j.canep.2018.10.002 30359894

[13] Zhang B, Kirov S, Snoddy J. WebGestalt: an integrated system for exploring gene sets in various biological contexts. Nucleic Acids Res. 2005;33(Web Server issue):W741–8. 10.1093/nar/gki475 PMC1160236 15980575

[14] Liu J, Shen JX, Wu HT, Li XL, Wen XF, Du CW, et al. Collagen 1A1 (COL1A1) promotes metastasis of breast cancer and is a potential therapeutic target. Discov Med. 2018;25(139):211–23. 29906404

[15] Kuivaniemi H, Tromp G. Type III collagen (COL3A1): Gene and protein structure, tissue distribution, and associated diseases. Gene. 2019;707:151–71. 10.1016/j.gene.2019.05.003 PMC6579750 31075413

[16] Gan YB. Expression of type I and type III procollagen genes in human scirrhous carcinoma of breast by in situ hybridization. Zhonghua Zhong Liu Za Zhi. 1992;14(5):348–50. 1337890

[17] Wang R, Fu L, Li J, Zhao D, Zhao Y, Yin L. Microarray analysis for differentially expressed genes between stromal and epithelial cells in development and metastasis of invasive breast cancer. J Comput Biol. 2020;27(12):1631–43. 10.1089/cmb.2019.0154 32429691

[18] Xiong G, Deng L, Zhu J, Rychahou PG, Xu R. Prolyl-4-hydroxylase α subunit 2 promotes breast cancer progression and metastasis by regulating collagen deposition. BMC Cancer. 2014;14:1. 10.1186/1471-2407-14-1 PMC3880410 24383403

[19] Chen Y, Pan Y, Ji Y, Sheng L, Du X. Network analysis of differentially expressed smoking-associated mRNAs, lncRNAs and miRNAs reveals key regulators in smoking-associated lung cancer. Exp Ther Med. 2018;16(6):4991–5002. 10.3892/etm.2018.6891 PMC6257755 30542454

[20] Srour MK, Gao B, Dadmanesh F, Carlson K, Qu Y, Deng N, et al. Gene expression comparison between primary triple-negative breast cancer and paired axillary and sentinel lymph node metastasis. Breast J. 2020;26(5):904–10. 10.1111/tbj.13684 PMC7211544 31713298

[21] Liu S, Liao G, Li G. Regulatory effects of COL1A1 on apoptosis induced by radiation in cervical cancer cells. Cancer Cell Int. 2017;17:73. 10.1186/s12935-017-0443-5 PMC5534093 28775672

[22] Guo Y, Zhai J, Zhang J, Ni C, Zhou H. Improved radiotherapy sensitivity of nasopharyngeal carcinoma cells by miR-29-3p targeting COL1A1 3′-UTR. Med Sci Monit. 2019;25:3161–9. 10.12659/MSM.915624 PMC6503752 31034464

[23] Shen H, Li L, Yang S, Wang D, Zhong S, Zhao J, et al. MicroRNA-29a contributes to drug-resistance of breast cancer cells to adriamycin through PTEN/AKT/GSK3β signaling pathway. Gene. 2016;593(1):84–90. 10.1016/j.gene.2016.08.016 27523474

[24] Li ZH, Xiong QY, Xu L, Duan P, Yang QO, Zhou P, et al. miR-29a regulated ER-positive breast cancer cell growth and invasion and is involved in the insulin signaling pathway. Oncotarget. 2017;8(20):32566–75. 10.18632/oncotarget.15928 PMC5464809 28427228

[25] Wu SY, Wu AT, Yuan KS, Liu SH. Brown seaweed fucoidan inhibits cancer progression by dual regulation of mir-29c/ADAM12 and miR-17-5p/PTEN axes in human breast cancer cells. J Cancer. 2016;7(15):2408–19. 10.7150/jca.15703 PMC5166552 27994679

[26] Li W, Yi J, Zheng X, Liu S, Fu W, Ren L, et al. miR-29c plays a suppressive role in breast cancer by targeting the TIMP3/STAT1/FOXO1 pathway. Clin Epigenetics. 2018;10:64. 10.1186/s13148-018-0495-y PMC5956756 29796115

